# Supplementary material for: Individual Radiosensitivity as a Risk Factor for the Radiation-Induced Acute Radiodermatitis
Source: Life (Basel). 2021 Dec 23;12(1):20. doi: 10.3390/life12010020 (PMC8781761; doi:10.3390/life12010020)
Supplement: Supplementary file 1 [file life-12-00020-s001.zip › life-1451308-supplementary.pdf]

Supplementary data

Table S1. Acute radiation dermatitis according to Common Terminology Criteria for Adverse Events scale (CTCAE), version 4.0.

Adverse Event	Grade				
	1	2	3	4	5
**ARD**	Faint erythema or dry desquamation	Moderate to brisk erythema; patchy moist desquamation, mostly confined to skin folds and creases; moderate edema	Moist desquamation in areas other than skin folds and creases; bleeding induced by minor trauma or abrasion	Life-threatening consequences; skin necrosis or ulceration of full thickness dermis; spontaneous bleeding from involved site; skin graft indicated	Death

ARD - Acute radiation dermatitis.

High inter and intravariability of radiation induced chromatid break in G2 assay was widely reported therefore classification of cases based on conventional G2 assay as well as reproducibility of results is hardly achievable. This study employs the methodology proposed by Pantelias, G.E et all in 2011 where applied caffeine-based abrogation of G2 checkpoint and comparison of G2 checkpoint efficiency within particular individual allows mitigating an impact of these variations into the evaluation of radiosensitivity factors. The main idea of the suggested adjustment of G2 assay is to mimic Ataxia telangiectasia syndrome (AT) and compare the efficiency of ATM reparation within a single patient when this pathway is "on" and caffeine-induced "off". Two chromosome aberration yields were set for each individual: standard G2 yield (caffeine-free) and next AT imitating high radiosensitivity level yield based on a caffeine-induced G2-M checkpoint arrest. IRS was considered as the ratio of standard G2 yield to imitated high radiosensitivity level in case of AT in each patient expressed in percentage. The evidence of the appropriateness of this kind of modification of G2 methodology is described in details in Pantelias, G.E. and G.I. Terzoudi, A standardized G2-assay for the prediction of individual radiosensitivity. Radiotherapy and Oncology, 2011. 101(1): p. 28-34. Table 1 depicts an example of intervariability in chromatid breaks yield expressed in our sample and how it was subtracted by suggested adjusted procedure via caffeine induced suppression of ATM and comparison of aberration yield in caffeine-free and caffeine-containing samples in the same patient: According conventional G2 assay patient Nr. 12 (292 aberration/50 cells) is less sensitive than patient Nr. 1 (322 aberration) but according to modified adjusted G2 assay it is vice versa Nr.12 (69 %) is more sensitive than Nr. 1 (58 %). This is also true regarding patients Nr. 2 and Nr 19: under conventional conditions of G2 assay Nr 2 seems to be more sensitive than Nr. 19 and in adjusted test it goes in opposite way and etc.

Table S2. Number of chromatid breaks and gaps per 50 cells according to conventional and modified G2 assays.

Number of Chromatid Breaks and Gaps per 50 Cells				
**No.**	**G2 yield**	**G2+Caffeine yield**	**IRS**	**Category**
1	322	551	58.44%	sensitive
2	445	660	67.42%	sensitive
3	403	539	74.77%	hypersensitive
4	427	518	82.43%	hypersensitive
5	537	627	85.65%	hypersensitive
6	509	694	73.34%	hypersensitive
7	400	652	61.35%	sensitive
8	418	641	65.21%	sensitive

9	308	435	70.80%	hypersensitive
10	276	468	58.97%	sensitive
11	441	560	78.75%	hypersensitive
12	292	421	69.36%	sensitive
13	280	586	47.78%	normal
14	303	575	52.70%	sensitive
15	294	474	64.92%	sensitive
16	311	517	60.15%	sensitive
17	328	501	65.47%	sensitive
18	376	474	79.32%	hypersensitive
19	300	349	85.96%	hypersensitive

G2 yield—conventional G2 assay; G2 + Caffeine yield—modified G2 assay; IRS—individual radiosensitivity; Category—IRS category.

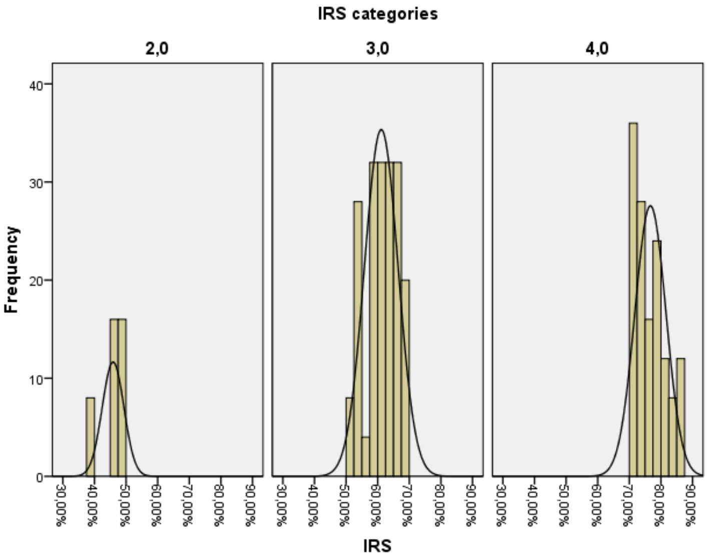

Figure S1. Histograms of IRS distribution in normal IRS, radiosensitive and Highly Radiosensitive patients groups. IRS—individual radiosensitivity; 2.0–(30% ≤ IRS ≤ 50%); 3.0–(>50% ≤ IRS ≤ 70%); 4.0–(IRS > 70 %).
